# Hospitals for the Paralysed

**Published:** 1907-11-23

**Authors:** 


### HOSPITAL ADMINISTRATION.

### CONSTRUCTION AND ECONOMICS.

### HOSPITALS FOR THE PARALYSED.

THEIR PRESSING NEED FOR MORE MONEY.

Busy Workers in Modern Cities.

Can anything surpass in pathos the fate of a man who, through a stroke of paralysis, is doomed to end a busy, active life in a state of absolute and entire dependence upon others? This question is one which directly concerns every brain-worker in the City of London, especially at the present time. The strain upon the most engaged of City men is often greater than any man ought in reality to face. In times of depression when, as in the last few years, each day may add to the weight of care the atmosphere of crisis or the absence of legitimate business, the pressure becomes aggravated, and the risk to health and even to life may thereby be great. It is true that large fortunes have been, and will continue to be, made in the City, but the struggle for existence, aggravated as it is by present conditions, and the prolonged burden of care which numbers have to bear, have tended to multiply the number of individual cases where the inevitable breakdown in health has occurred.

Is it to be wondered in such circumstances that the facts should show a very large increase in the growth of cases of paralysis, as represented by the actual patients under treatment at the Metropolitan hospitals for the paralysed and epileptic? Thus, the number of patients treated at the National Hospital for the Paralysed in twenty years has increased from 3,561 in 188[illegible] to 7,746 in 1906—that is, by upwards of 100 per cent. Unfortunately, although the cost of providing for these cases of paralysis has also increased by nearly 100 per cent.—that is, from £8,786 to £17,062—the total income available has only increased by under 60 per cent., *i.e.* from £7,557 to £12,711. Twenty years ago there was only one hospital for the paralysed; now there are three. In 1896 these three hospitals treated 11,268 patients, costing £18,270, and the numbers had grown by the end of 1906 to 14,552 patients, costing £25,216. Once again, unfortunately, the income of the three hospitals, which was £13,066 in 1896, has only increased to £19,186 in 1906, leaving a deficiency in revenue to defray the cost of treating cases of paralysis of upwards of £6,000 a year. We commend these figures to the attention of all who take an intelligent interest in the medical requirements and needs of the people of London, seeing that the increase in cases of paralysis, though it applies to all classes, must necessarily apply mostly to the keenest workers in our midst, who bear most of the strain and stress of the higher and more exacting work, which, collectively, represents the advancement or decline of the nation's progress.

Taken as a whole, there are probably no better-administered hospitals than those for paralysis. There is certainly no class of hospital more needed, or where further accommodation is more urgently called for. It is false economy not to encourage science to attain the highest developments in regard to diseases which are increasing, especially as they affect chiefly those lives which are most valuable to the whole community for the reasons already stated.

In view of the facts we have given we venture to address a most urgent appeal to the City of London, as well as to all wealthy people, who, in addition to wealth, have also a measure of the higher intelligence, for then, we are certain, our hospitals for nervous diseases and paralysis will speedily be placed in a sound financial position. Make the hospitals for paralysis prosperous, and the community will show its wisdom by effecting a general insurance of the utmost value to the progress of the nation. There never was a time when everything which the highest medical intelligence and scientific knowledge can provide for the protection of the strenuous worker was more needed than it is to-day, having regard to the actual position of business which has prevailed in the City of London during recent years, and which continues at the present time.

The coming Jubilee of the National Hospital for the Paralysed and Epileptic is an occasion which the public should welcome, as it will afford them an excellent opportunity to show that they apprehend the importance of the considerations we have just urged in favour of much more liberal pecuniary support for hospitals of this character. The following account of a meeting held in Queen Square on the 19th instant cannot fail to be read with special interest in the circumstances:—

#### THE HOSPITAL FOR THE PARALYSED AND EPILEPTIC.

The Coming Jubilee.

A meeting of Governors and members was held at the National Hospital for the Paralysed and Epileptic, Queen Square, Bloomsbury, on Tuesday afternoon, in order to discuss the best means of celebrating the jubilee of the institution in 1909. Her Royal Highness the Duchess of Albany attended the meeting, and there were also present Mr. Frederick Macmillan, Chairman, Sir John Paget, K.C., Vice-Chairman, Mr. William Crowe, Sir Felix Semon, Mr. Coningsby Disraeli, Mr. Arnold Royle, C.B., and Dr. Risien Russell.

The Chairman referred to the origin of the Hospital nearly fifty years ago, when, early in November 1859, Mr. and the Misses Chandler, at a meeting in the Mansion House, proposed that a Home for Paralysed Women should be started. In this form the Hospital was carried on in private houses on its present site for a quarter of a century, and the freehold being acquired in 1880 some of the existing buildings were erected. The late Duke of Albany was greatly interested in the institution, and a portion of it is a memorial to him. It was now proposed that, to celebrate the jubilee, the following scheme should be carried out:—The extension of the out-patients' department by 520 square feet, with new entrance in Powis Place; two new and larger consulting rooms, each with two dressing rooms; new inquiry office; new and enlarged dispensary; new ophthalmic room; twenty-five additional bedrooms for sisters and female nurses; new dining-room, night nurses' sitting-room, and bath-rooms; new physical exercise room; new class-room for the Massage and Electrical Schools; new staff dining-room; and several minor improvements. This scheme, it was estimated, would cost 10,000*l.*, but it would not increase the current expenses of the Hospital as the erection of new wards would have done. The difficulty, of course, was to find the money; but by great good fortune a lady, closely connected with the Hospital, had offered to raise the sum required, so long as she might work in her own way, form a committee, and preside over it herself. This lady was Her Royal Highness the Duchess of Albany. He submitted a resolution approving the scheme, respectfully asking Her Royal Highness to form a committee to raise the necessary funds.

Dr. T. Buzzard seconded.

Dr. Danvers Power supported the resolution, and it was carried with enthusiasm.

Speech by the Duchess of Albany.

The Duchess of Albany said that she willingly accepted the office of President of the Committee. She hoped that they would be able to fulfil their purpose, and she would do her best loyally to aid them if they did not mind assenting to her suggestions. She was very anxious that not one penny of expenses in connection with the work of the jubilee should fall on the Hospital, so that the committee might be able to appeal to the public with a straight promise that every contribution, however small or large, should go to the Hospital without any deduction. Before they could begin work it was therefore essential to open an expense fund—not a guarantee fund—any surplus of which would ultimately be handed over to the Hospital. Towards this expense fund the Chairman had given her 100*l.* as a start, and she was sure that the Secretary would be very pleased to take down any more names. She would be glad to contribute her own mite. She had already in hand 112*l.* for the jubilee fund. She began work on November 4; by the 8th she had received two cheques, and several promises, and she hoped that this was an augury that the Hospital would ultimately receive all that she wanted it to have.

A vote of thanks to the Duchess of Albany closed the proceedings.

